# COVID-19 in People With Schizophrenia: Potential Mechanisms Linking Schizophrenia to Poor Prognosis

**DOI:** 10.3389/fpsyt.2021.666067

**Published:** 2021-05-17

**Authors:** Mohapradeep Mohan, Benjamin Ian Perry, Ponnusamy Saravanan, Swaran Preet Singh

**Affiliations:** ^1^Division of Mental Health and Wellbeing, Warwick Medical School, University of Warwick, Coventry, United Kingdom; ^2^Department of Psychiatry, University of Cambridge, Cambridge, United Kingdom; ^3^Populations, Evidence and Technologies, Division of Health Sciences, Warwick Medical School, University of Warwick, Coventry, United Kingdom; ^4^Academic Department of Diabetes, Endocrinology and Metabolism, George Eliot Hospital, Nuneaton, United Kingdom; ^5^Coventry and Warwickshire Partnership Trust, Coventry, United Kingdom

**Keywords:** schizophrenia, COVID-19, severe mental illness, SARS-CoV-2, first episode psychosis, psychosis

## Abstract

As the global burden of mortality from COVID-19 continues to rise, an understanding of who is most at risk of adverse outcomes is of paramount importance. Pre-existing cardiometabolic, renal and respiratory diseases as well as old age are well-established risk factors associated with disease severity and mortality among patients with COVID-19. However, mounting evidence also indicates an increased susceptibility to, and risk of adverse outcomes from COVID-19 in people with schizophrenia, independent of age and comorbidity. Therefore, elucidating the underlying pathophysiological mechanisms which may increase the risk of poor outcomes in people with schizophrenia is of crucial importance. Here, we provide a narrative on the current understanding of COVID-19 in patients with schizophrenia and propose potential mechanisms which may link schizophrenia with an increased susceptibility to, and greater risk of adverse outcomes from COVID-19. Given the existing knowledge gaps, robust clinical and biological studies are required to further our understanding of some of these underlying mechanisms, so that effective prevention and treatment strategies for COVID-19 in patients with schizophrenia can be developed.

## Introduction

The novel coronavirus disease-2019 (COVID-19), caused by the highly infectious severe acute respiratory coronavirus 2 (SARS-CoV-2), has led to more than 2 million deaths globally as of March 2021 (1), and the global mortality continues to rise. The clinical manifestations of SARS-CoV-2 infection are broad, ranging from asymptomatic or mild flu-like symptoms, through to fatal outcomes including respiratory syndromes requiring mechanical ventilation, multiorgan failure and death (2, 3). Epidemiological studies have reported a greater risk of adverse clinical outcomes of COVID-19 in men, the elderly, and in those with pre-existing comorbidities including cardiovascular diseases (CVD), diabetes mellitus (DM), renal and respiratory diseases (4). In addition, a robust body of evidence suggest that the determinants of severity of SARS-CoV-2 infection are more likely to be related to host factors, rather than the viral genetic variation (3).

Whilst global attention has largely focused on COVID-19 patients with significant physical comorbidities, the clinical impact of COVID-19 on people with severe mental disorders (SMD) such as schizophrenia has been relatively overlooked. Predictors of disease severity in COVID-19 such as CVD, DM, chronic obstructive pulmonary disease (COPD) and chronic immune dysregulation are more prevalent in people with schizophrenia, than the general population (5–11), suggesting the possibility of a more pronounced severity of COVID-19 in people with schizophrenia. Furthermore, people with schizophrenia may also be at increased risk of contracting COVID-19 due to the chronic nature of their illness, behavioural factors relating to cognitive impairment and poor risk awareness, sociodemographic characteristics including homelessness and congregate housing and the adverse effects of antipsychotics (12). In line with these observations, emerging epidemiological evidence suggests that people with schizophrenia could be at increased risk of COVID-19 and its adverse outcomes (see Table 1). However, it remains unclear whether schizophrenia itself independently increases the risk of severe COVID-19 infections or whether concomitant comorbidities and risk factors may be driving such worse prognosis.

**Table 1:** COVID-19 outcomes according to pre-existing schizophrenia.

**References**	**Article type**	**Study population**	**Prevalence of schizophrenia**	**Outcome**	**Risk**
Fond et al. (13)	Cohort study	50,750	1.60%	In-hospital mortality	1.30 (1.08–1.56)^*^
Fond et al. (13)	Cohort study	50,750	1.60%	ICU admission	1.58 (1.09–2.3)^*^
Lee et al. (14)	Cohort study	7,160	NA	SARS-CoV-2 infection	1.00 (0.93–1.08)^*^
Lee et al. (14)	Cohort Study	7160	NA	Composite	2.27 (1.50–3.41)^*^
Wang et al (15).	Retrospective	61,783,950	NA	SARS-CoV-2 infection	7.34 (6.65 −8.10)^*^
Ji et al. (16)	Retrospective	219,961	3.6%	SARS-CoV-2 infection	1.6–1.7 (ORR)
Nemani et al. (17)	Retrospective	7,348	1%^**^	In-hospital mortality	2.67 (1.48–4.8)^*^
Taquet et al. (18)	Retrospective	62,354	2%	SARS-CoV-2 infection	1.17 (1.02–1.33)^*^

*Composite, Death or ICU admission or invasive ventilation; ICU, Intensive care unit; NA, Not given;*

* *Odds ratio (95% CI);*

** *Schizophrenia spectrum disorder; ORR, Odds ratio range (95% CI not given)*.

In this article, we aim to: (i) appraise the available epidemiological evidence of COVID-19 in people with schizophrenia; and (ii) narratively describe and integrate, as a hypothesis, the available evidence on the potential pathophysiological underpinnings that may confer vulnerability to severe SARS-CoV-2 infection in schizophrenia. Based on the current understanding of clinical features of the virus, we hypothesise that people with schizophrenia are at increased risk of severe SARS-CoV-2 infection and heightened risk of mortality, irrespective of baseline comorbidities, treatment and lifestyle factors. Insights into the biological mechanisms that contribute to to COVID-19 susceptibility and prognosis will be crucial in development of new therapeutic targets, furthering our knowledge of tissue tropism, and will also help tailoring public health strategies relevant to this group of patients. We recommend that people with schizophrenia should be considered a priority group in vaccine allocation.

The psychosocial impact of COVID-19 pandemic on people with schizophrenia has been described elsewhere (12, 19, 20), and is beyond the scope of this article.

## Methodology

For this article, PubMed and Google Scholar were searched for published or unpublished articles, up to 18th March, 2021, using the search terms “schizophrenia,” “psychosis,” “first episode psychosis,” “SARS-CoV-2,” “COVID-19,” “coronavirus,” “mental illness,” “mental disorders,” “severe mental illness/disorders, “obesity,” “antipsychotics,” “inflammation,” “outcome,” “comorbidities,” “age', “gender', “ethnicity,” “mendelian randomisation,” “neurotropism,” “central nervous system,” “ACE 2,” “renin-angiotensin system,” “pathophysiology” and a combination thereof. We incorporated peer-reviewed articles and two preprint articles. We also referred to related scientific reports, such as the Care Quality Commission, UK, and Public Health England guidance on clinically extremely vulnerable groups (CEV) for vaccine prioritisation. Formal quality assessment of studies were not undertaken for this hypothesis article, nor was a systematic review or meta-analysis conducted.

## Interaction Between COVID-19 and Schizophrenia: Epidemiological Evidence

People with schizophrenia are known to suffer from a higher prevalence of respiratory tract infections than general population (21–23). The global age-standardized prevalence of schizophrenia is estimated to be 0.28% (24). The prevalence of schizophrenia in 7341 Korean patients with COVID-19 was 3.6% (16), over five times higher than the national prevalence of schizophrenia in Korea (0.66%) (25). Importantly, the prevalence of schizophrenia in severe COVID-19 was even higher, at 5.4% (16). In 50,750 patients admitted to hospital for COVID-19 in France, the prevalence of schizophrenia was 1.6%. (13), which is 60% higher than the lifetime prevalence of schizophrenia in the West. A retrospective study from US reported a prevalence of 2% for psychotic disorders in 62,354 patients with COVID-19 (15).

Schizophrenia may confer an inherent risk for COVID-19. A recent large retrospective cohort study reported that psychotic disorders (including schizophrenia) could be an independent risk factor for COVID-19 (Risk ratio RR 1·17 95% CI 1·02–1·33; *p* = 0·022), irrespective of physical risk factors for COVID-19 and duration of psychotic illness (18). A case-control study from South Korea reported an increased risk of COVID-19 in patients with schizophrenia (odds ratio range, 1.614–1.721) (16). Similar observations were made in another case-control study from US, which reported an increased risk of COVID-19 among patients with schizophrenia, after adjusting for potential confounders (adjusted odds ratio AOR = 7.34, 95% CI: 6.65–8.10, *p* < 0.001) (15). There is also some evidence of rapid SARS-CoV-2 transmission in psychiatry units (26, 27). The heightened risk may be specific to schizophrenia rather than generalised across all mental disorders. For instance, a nationwide South Korean cohort study reported that diagnosis of any mental illness was not associated with higher rates of SARS-CoV-2 infection (AOR 1.00, 95% CI 0.93–1.08) (14). A recent mendelian randomisation (MR) study suggests a significant genetic liability to a combined phenotype of bipolar disorder and schizophrenia with increased COVID-19 susceptibility, but the effect estimates were not consistent in multivariate analysis (28). However, this should be interpreted with caution because of the usage of small sample size in the early genome-wide association studies of COVID-19.

Schizophrenia appears to be associated with poorer prognosis in patients with COVID-19. A French population—based cohort study has reported a higher odds of mortality among in-patients with schizophrenia compared with controls [AOR 1.30 [95% CI, 1.08–1.56], *P* = 0.0093) (13). Another case-control study reported that patients with SMD including schizophrenia are 1.3 times more likely to experience severe outcomes (mortality, or invasive ventilation or admission to ICU) from COVID-19 (AOR 1·3, 95% CI 1·0–1·7) (15). A propensity score matched nationwide cohort study reported that patients with SMD including schizophrenia are more likely to experience severe COVID-19 outcome (ICU admission, invasive ventilation, or death) compared to those without mental illness (AOR 2.27, 95% CI 1·50–3.41) (14). A separate case-control study also reported that schizophrenia was independently associated with severe SARS-COV-2 infection (odds ratio range, 1.206–1.645), further suggestive of an intrinsic link between schizophrenia and SARS-CoV-2 infection (16). Furthermore, a recent retrospective cohort study reported higher risk of mortality [[OR], 2.67; 95% CI, 1.48–4.80] in patients with schizophrenia spectrum disorder (17). Strikingly, in this study, schizophrenia was reported to be the second-highest risk factor for COVID-19 mortality, after age, followed by other comorbidities such as heart failure [OR = 1.60, 95% CI (1.43–2.00)], hypertension [OR = 1.38, 95% CI (1.12–1.70)], diabetes [OR = 1.27, 95% CI, (1.07–1.51)] and cancer [OR = 1.01, 95% CI, (0.85–1.22)]. It is noteworthy that all the study estimates described above were adjusted for potential confounders, including age, gender and chronic medical comorbidities such as CVD, DM, COPD, cancer etc. Finally, the UK Care Quality Commission has also highlighted higher COVID-19 related death rates in institutionalised populations with mental illness (29). Together, these findings indicate that people with schizophrenia may carry both an inherent vulnerability toward COVID-19, and increased risk of more adverse clinical outcomes.

Given the relative novelty of the SARS-CoV-2 virus, unravelling and integration of biologically plausible links with clinical SARS-COV-2 data is crucial in order to improve our understanding of the pathophysiology of the disease.

## Schizophrenia and COVID-19: Prognostic Factors and Potential Mechanisms

### Age, Gender, and Ethnicity

The risk of SARS-CoV-2 infection and associated mortality increases with age, with males more at risk of higher mortality rates than females (30). The average age of patients with schizophrenia hospitalised for COVID-19 is younger than the general population (13). Moreover, a recent study has reported that younger patients with comorbid schizophrenia (<55 years) and COVID-19 had an increased risk of intensive care unit (ICU) admissions (13). In addition, age may also interact with schizophrenia liability for increased risk of SARS-CoV-2 infection. A recent case-control study reported that older adults with schizophrenia (>65 years) had a higher risk of SARS-CoV-2 infection than those aged 18–65 years (adjusted OR 1.74, 95% CI:1.33–2.28, *p* < 0.001) (15).

The prevalence of COVID-19 in people with comorbid schizophrenia is not dissimilar between the genders (49 and 51% for males and females, respectively), while women had higher death rates and less ICU admissions than men (13). This is discrepant to the general population, where male gender is a risk factor for ICU admission and mortality (31). In another study, women with schizophrenia had higher incidence of SARS-CoV-2 infection than men (adjusted OR = 1.53, 95% CI: 1.21–1.94, *p* < 0.001) (15).

In the general population, mortality rates from SARS-CoV-2 infections among people from ethnic minorities are two to four times higher than in the White European population (32). These findings are echoed in the schizophrenia population. A recent case-control study reported that, as compared to White patients, African Americans with schizophrenia had higher prevalence of SARS-CoV-2 infection (adjusted OR: 2.33, 95% CI 1.84–2.97, *p* < 0.001 and higher mortality rates (6.2 vs. 3.7%, *p* < 0.001), with men having higher mortality rates than women (6.6 vs. 3.4%, *p* < 0.001) (15).

### Comorbidities

The risk factors for severe SARS-CoV-2 infection such as CVD, DM and chronic respiratory diseases such as COPD (4, 33), are more common in people with schizophrenia than in the general population (5, 6, 8). In patients tested positive for SARS-COV-2, people with mental illness including schizophrenia had a greater prevalence of concomitant medical comorbidities compared with those without mental illness (diabetes 27.3 vs. 9.7%; CVD 15.5 vs. 4.9%; cerebrovascular disease 16.6 vs. 3.8%; COPD 11.2 vs. 3.3%; asthma 16 vs. 8.4%; hypertension 43.2 vs. 17.8%; chronic kidney disease 5.8 vs. 3%) (14). A similar trend in comorbidities (such as CVD, COPD, cancer, chronic kidney disease, and asthma) was reported in another study (15). Importantly, the increased risk of COVID-19 in patients with SMD remained after adjusted analysis, suggesting that the underlying SMD itself could directly contribute to the increased risk of COVID-19 infections (15). In hospitalised COVID-19 patients with schizophrenia, the prevalence of CVD was (23%), cerebrovascular disease (8%), COPD (15%), diabetes (25%), dementia (25%), cancer (6%), and renal disease (12%) compared to those without schizophrenia (29, 6, 13, 28, 9, and 14%, respectively) (13). The prevalence for CVD was higher among younger patients (<55 years) who also had more ICU admissions (13). Moreover, in people with schizophrenia, non-survivors had greater prevalence of comorbidities than survivors (renal disease 20.3% vs. 9.8%; hemiplegia 5.4 vs. 3.9%; peripheral vascular disease 9.2 vs. 4.7%; Metastases 6.2 vs. 2.1%; Cancer 15.1 vs. 7.1%; diabetes with complication 9.3 vs. 5.3%; diabetes without complications 25.6 vs. 20.6%; dementia 16.7 vs. 7.9%; cerebrovascular disease 10.1 vs. 5.2%; COPD 14.8 vs. 12.1%; congestive heart failure 27.4 vs. 13%; myocardial infarction 12 vs. 6.1%) (13). All these studies reported a greater risk of mortality after adjusting for potential cofounders (including age, gender and physical comorbidities), suggesting that schizophrenia may independently contribute to poor prognosis in COVID-19.

### Pathological Immune Response in Schizophrenia and COVID-19 Infections

A potential mechanism linking schizophrenia to more severe SARS-CoV-2 infection is the dysregulated immune system (34–36). Circulating levels of inflammatory biomarkers (including interleukin-6 (IL-6), tumor necrosis factor alpha (TNF-α), C-reactive protein (CRP), growth differentiation factor 15 (GDF-15) and D-dimer are often significantly elevated in severe SARS-CoV-2 infection and have been associated with increased risk of mortality (34, 37, 38). In COVID-19, this cytokine overproduction can lead to cytokine storm leading to adverse clinical outcomes such as multiorgan failure and deaths (39).

Several studies have reported that, both chronic schizophrenia and drug-naive first-episode psychosis (FEP) are characterised by a systemic pro-inflammatory state, as evidenced by higher levels of proinflammatory biomarkers such as IL-1β, IL-6, TNF-α, GDF-15, and CRP (7, 40–45). Importantly, high levels of pro-inflammatory cytokines have been reported in the cerebrospinal fluid and serum of people with schizophrenia (46), implicating that inflammatory changes measured in the periphery may mirror those found in CNS. In addition, various neurological manifestations have also been implicated in SARS-CoV-2 infections that can result in a dysregulated CNS immune response (47, 48). This neurotropism of SARS-CoV-2 is often attributed to structural and functional brain changes, especially glial cells and astrocytes, which orchestrate innate immunity in the brain (49). Glial cell dysfunction (50–52), particularly chronic activation of microglial cells (53), is a common feature in the pathophysiology of schizophrenia, and is associated with deleterious proinflammatory effects (53).

Given the central role of immune dysregulation in developing fatal SARS-CoV-2 infections, and that schizophrenia is already characterised by subclinical inflammation, the systemic hyper-inflammation triggered by SARS-CoV-2 infection may be more pronounced in people with schizophrenia. These exacerbated proinflammatory responses can trigger systemic tissue damage and multiorgan failure. It is to be noted that high concentrations of inflammatory biomarkers such as CRP (>200 mg/L) and ferritin (>2,500 ng/mL) at admission have been found to be strong predictors of COVID-19 severity (54). Similar conclusions can also be drawn from a recent study, where patients with SMD including schizophrenia who were hospitalised for severe COVID-19 had high levels of inflammatory biomarkers such as CRP, IL-6, D-dimer and ferritin, on admission (54, 55). Furthermore, a relationship exists between lymphocyte subpopulations such as the natural killer (NK) cells that contribute to early antiviral immunity, and schizophrenia. Natural killer cell depletion is a common finding both in people with schizophrenia (56) and in severe COVID-19 (57). Higher viral loads can attenuate the immune response in people with schizophrenia due to reduced NK activity, exposing them to more severe infection. This might, at least in part, help to explain why people with schizophrenia are more susceptible to COVID-19 and experience worse clinical outcome, as compared to general population. Taken together, these findings imply that changes in pathways which regulate immunomodulation could be one of the mechanism linking schizophrenia and severe COVID-19. Since people with schizophrenia are already in a state of hyperinflammation, a profound inflammatory response to SARS-CoV-2 infection could trigger adverse clinical outcomes and contribute to an increased risk of mortality from COVID-19.

### Antipsychotic Use and Immunodeficiency

Another potential mechanism linking schizophrenia with an increased risk of poor COVID-19 clinical outcomes might be attributable to the use of antipsychotics. There is some evidence to suggest that exposure to antipsychotic therapy is associated with increased risk of respiratory infections such as pneumonia, and thromboembolism (58, 59), both of which are fatal clinical manifestations of COVID-19 (60). Antipsychotics, especially clozapine are associated with an increased risk of agranulocytosis and blood dyscrasias, and therefore an increased vulnerability to infections (61, 62). A few studies have investigated the effects of antipsychotic prescribing on COVID-19 outcomes. A recent retrospective cohort study reported that clozapine was associated with increased risk of SARS-CoV-2 infection compared with other antipsychotic medications (adjusted HR = 1.76, 95% CI 1.14–2.72) (63). A recent systematic review recommended careful monitoring of clozapine in COVID-19 patients (64). Another atypical antipsychotic, risperidone has been shown to be associated with global immunosuppression and increased susceptibility to infections (65). While the clinical data regarding the safety of risperidone in COVID-19 is sparse, preclinical studies have reported a direct deleterious effect of risperidone on inflammatory and immune process regulation (66). In the premise of inflammatory storm and consequent higher mortality rates in COVID-19, this observation may be worth considering in risk prognostication. Furthermore, recent reports on haloperidol on COVID-19 outcomes are varied. An observational study reported no association between haloperidol prescribing and risk of intubation, death or discharge time among patients hospitalised for COVID-19 (50). In contrast, neuroleptic malignant syndrome, which is typically associated with antipsychotics, has been reported in a case-study of a middle aged male with comorbid schizophrenia and severe COVID-19, who was prescribed haloperidol (51). In addition, COPD is very common in patients with schizophrenia (8), and antipsychotic use is associated with an increased risk of acute respiratory failure in people who have COPD (52). Furthermore, antipsychotics have been shown to be associated with an elevated risk of thrombotic events (58, 67). COVID-19 is associated with coagulation abnormalites, that can lead to fatal thromboembolic events (68–71). Therefore, once infected, the risk of thromboembolic events may be more pronounced in people prescribed antipsychotics. The evidence to date on the association between antipsychotics and COVID-19 are predominantly from observational studies and case reports, therefore further studies are warranted to clarify whether antipsychotics could have a detrimental effect on the SARS-CoV-2 clinical course. However, based on our knowledge on the pharmacology of antipsychotics, and after taking into account of the evidence presented above, it is worth considering that some classes of antipsychotics may carry an increased risk of poor prognosis in patients with COVID-19. In line with this hypothesis, a recent review of evidence by an international, multi-disciplinary working group of experts reported that antipsychotics may carry an increased risk of worsening respiratory function and thromboembolism in patients with COVID-19, and proposed practical recommendations for the clinical management of psychotropics in patients with COVID-19 (72).

### Obesity

Obesity, especially abdominal obesity, is a well-acknowledged comorbidity among patients with chronic schizophrenia (7, 73), and is a strong risk factor for susceptibility, transmission and severity of COVID-19 (74, 75). In a recent meta-analysis of COVID-19 studies, obesity was associated with high morbidity and mortality rates, and increased ICU admission rates (76). Pertinent to these observations, a recent study reported that obese patients with comorbid schizophrenia had 14% more ICU admissions than controls (13). Several mechanisms can proposed by which obesity could contribute to increased susceptibility, transmission and poor COVID-19 outcome in people schizophrenia.

First, as mentioned earlier, obesity is highly prevalent in people with chronic schizophrenia (8), and ACE2 gene expression has been reported to be higher in visceral and subcutaneous adipose tissue than in lung tissues (59). ACE2 is the principal cellular entry receptor for SARS-CoV-2 (77), and this mechanism may suggest a high viral propagation in people with schizophrenia who are obese (78). The adipose tissue may, therefore act as a viral reservoir, contributing to prolonged viral shedding (79), and a more contagious state in people with schizophrenia. Second, both schizophrenia and obesity are characterised by dysregulated immune response to infections, contributing to increased susceptibility to infections (34–36). The immunomodulatory effects of obesity that can impair key immune response to SARS-CoV-2 is well-acknowledged (74). Third, obesity also contributes to diminished lung function and life threatening thrombolytic events in COVID-19 patients (74). Although old age is one of the major risk factors for severe SARS-CoV-2 infection, young individuals with schizophrenia who are also obese may be at risk of greater severity of COVID-19, than their peers. A recent study reported that schizophrenia patients under 55 years, who were predominantly obese had 14% higher risk for more ICU admissions than controls (13). Furthermore, individuals with obesity and schizophrenia have lower vitamin D concentrations, which could also impair immune response (80, 81). There is mounting evidence that vitamin D deficiency could be a risk factor for adverse COVID-19 outcomes (81), and the efficacy of vitamin D supplementation in reducing risk and/or severity of COVID-19 is currently being investigated in randomised controlled trials. Taken together, these findings suggest that obesity might also be a strong risk factor for adverse clinical outcomes in patients with schizophrenia.

### COVID-19, Coagulopathies, and Schizophrenia

In severe cases of COVID-19, fatal coagulation abnormalities that can be life-threatening, has been widely reported (68–71). More recent researches have highlighted the coagulation cascade in COVID-19 through mechanisms (possible synergistic) such as endothelial dysfunction, systemic inflammation, tissue damage, platelet activation and hypoxic vasoconstriction, resulting in fatal thromboembolic events (82). Moreover, impaired fibrinolysis, as evidenced by higher levels of plasminogen activator inhibitor (PAI-1), has been reported in patients with COVID-19, which could further elevate the thrombotic risk (83). Most patients with severe COVID-19 and coagulopathy presents with an increased concertation PAI-1 and D-dimer, which are also associated with fatal thrombotic complications (83, 84). Furthermore, anticoagulant therapy appears to confer prognostic benefit in severe COVID-19 patients (85).

There is evidence to suggest that schizophrenia may be a hypercoagulable state, due to the underlying inherent pathobiological process (86–88) and antipsychotic medication (67, 89, 90). Importantly, increased levels of PAI-1 have been reported in people with schizophrenia than controls (91), suggesting an imbalance between clotting factors and fibrinolysis. In a retrospective study from New York City, elevated levels of prothrombotic markers such as D-dimer was reported among hospitalised patients with severe COVID-19 and mental illness including schizophrenia (55). Given that coagulation abnormalities may be associated with both schizophrenia and SARS-CoV-2 infections, the coexistence of these conditions might confer a cumulative risk of thromboembolic events.

The precise mechanisms underlying hypercoagulation in patients with schizophrenia and COVID-19 are unclear. However, taking available evidence into account, we hypothesise that dysregulated immune response, obesity and endothelial damage/dysfunction, which are common in schizophrenia (92–94) and severe COVID-19 (95), might predispose people with schizophrenia to a higher risk of thromboembolic events, once infected with SARS-CoV-2. Taken together, it is reasonable to assess the risk of thromboembolic events and to consider thromboprophylaxis in people with schizophrenia, if they are hospitalised with COVID-19. Prospective studies are warranted to validate the efficacy of anticoagulation therapy in preventing thrombotic events in this group of patients.

### Non-diabetic Hyperglycemia

While underlying DM is considered a strong risk factor for increased COVID-19 disease severity and higher mortality (96), recent evidence suggest that prediabetes (non-diabetic hyperglycemia or intermediate hyperglycemia) is also associated with the severity of COVID-19. A prospective study from a large suburban hospital from New Jersey reported that in COVID-19 patients, prediabetes was independently associated with intubation (97). A retrospective study from China reported that impaired fasting glucose was an independent predictor for 28-days mortality in COVID-19 patients without DM (98). Similarly, another study of hospitalized COVID-19 patients, pre-existing prediabetes was associated with higher risk of mortality, compared with those with normoglycemia (99). Moreover, previous studies have reported a positive association between prediabetes and inflammatory biomarkers that are involved in the severity of COVID-19, such as CRP and IL-6 (100, 101).

The foregoing findings may carry important clinical implications in schizophrenia. There is evidence to suggest an intrinsic link between disrupted glucose-insulin homeostasis and schizophrenia. For instance, recent evidence from a population-representative birth cohort has found that disrupted glucose-insulin homeostasis from mid-childhood is longitudinally associated with psychosis in adulthood in a dose-dependent manner, even after adjustment for a detailed range of sociodemographic and lifestyle confounders. Furthermore, meta-analysis have reported that non-diabetic hyperglycemia (such as insulin resistance and prediabetes) is highly prevalent in antipsychotic naïve patients with schizophrenia, compared with matched controls (9, 102).

Both diabetes and prediabetes are characterised by impaired insulin secretion and insulin resistance (100). Notably, the main pathophysiological mechanisms implicated in the severity of SARS-CoV-2 infection, including dysregulated immune responses, innate immune defects, reduced adaptive immunity to infections and hypercoagulable state (11), also characterise both prediabetes (100), and schizophrenia, as stated already. Indeed, recent evidence from a Mendelian randomization study suggests that inflammation may be a common biological mechanism for comorbid insulin resistance and schizophrenia (103). Although prediabetes is not conventionally viewed as a comorbidity, preliminary evidence suggest that pre-diabetes could be a risk factor for severe COVID-19, and this risk may be magnified in people with schizophrenia as glucose homeostasis is impaired, even in drug naïve people with schizophrenia (102). Large studies are required to investigate this relationship further in the context of schizophrenia.

## Treatment for Early COVID-19 in Schizophrenia—A Critical Need

People with schizophrenia represent a highly vulnerable population, whose immune response may be diminished than general population. The early- onset infection with SARS-CoV-2 is purported to be mild (104), providing a potential window for early interventions, aimed at prevention of disease progression and reducing the risk of hospitalization and deaths. While there are effective therapeutic options available for moderately to severely ill hospitalized COVID-19 patients, such as remdesivir and dexamethasone (105, 106), effective treatment modalities for early COVID-19 (mild to moderate disease) have been more elusive. Remdesivir does not appear to offer any significant clinical benefit for patients with mild or moderately severe COVID-19 who are without respiratory support, and the National Institute for Health and Care Excellence (NICE) do not recommend its use in this group of patients (107). Dexamethasone is yet to be tested in early covid-19, and its immune-suppressive effects warrants caution when prescribed to people with schizophrenia, as it may inadvertently lead to poor treatment outcomes (108, 109). Due to the multifaceted pathophysiology of the SARS-CoV-2 virus, no single proven therapeutic agent exists to date. An ideal early therapeutic option in this group of patients may be the one that can address the principal components of infection severity that are shared between schizophrenia and COVID-19, such as cardiometabolic factors (e.g., obesity and prediabetes), hypercoagulable state, and inflammation (110). In this context, accruing evidence from pilot RCTs and observational studies have reported mortality benefits for antiplatelet and anticoagulant agents such as aspirin and heparin, non-steroidal anti-mitotic drug such as colchicine and oral hypoglycaemic agents such as metformin, in a diverse population with COVID-19 (110–112). All of these agents are already proven clinically safe and are in use for treating other conditions. However, rigorous well-powered clinical trials will be needed to ascertain the effectiveness of such treatments for patients with schizophrenia. Finally, it is important to consider potential drug-drug interactions (DDI) with COVID-19 medications and antipsychotics. Such DDIs can result in either reduced drug tolerance reduced efficacy or reduced safety. A recent systematic review involving three drug interaction databases reported risk of QT-prolongation and Torsade de Pointes, and cytochromes P450, as major interactions between COVID-19 drugs and antipsychotics (113). This systematic review suggests a high risk of DDI between lopinavir/ritonavir plus quetiapine and ritonavir/indinavir plus risperidone, hematological risk with clozapine and baricinitib, caution for favipiravir plus chlorpromazine and quetiapine, while no DDI risk was associated with remdesivir, baricinitib, tocilizumab and anakinra (113).

Irrespective of the proposed treatment regimen, given that COVID-19 is transmitted through droplet and aerosol spread, a period of self-isolation will always be required. Studies have shown that self-isolation can lead to substantial psychological burden in people with schizophrenia (114, 115). In addition, such self-isolation may impede the patient from receiving their usual antipsychotic treatment, particularly in instances where treatment is provider administered, such as with long acting injectable antipsychotics; or in instances where relatively intensive monitoring is required, such as with clozapine. Delays or disruption in receiving antipsychotic medications may increase the risk of psychosis relapse (116). The Royal College of Psychiatrists in the UK has now provided specific guidance regarding psychotropic treatment in people with a positive COVID-19 diagnosis (117).

## Discussion

**Figure 1:**
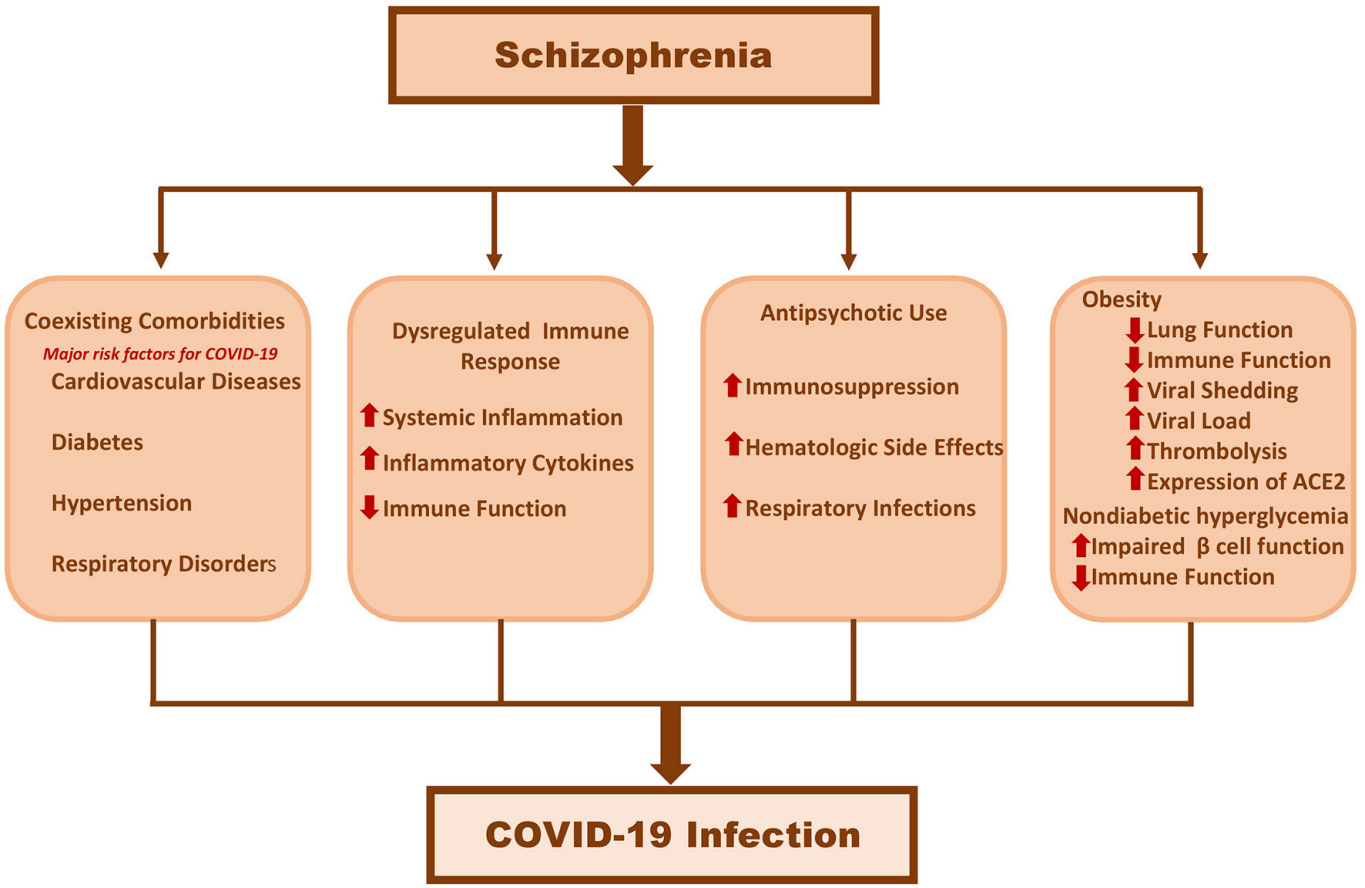
Hypothetical pathogenesis linking schizophrenia to severe Covid-19 infections.

In this article, we have summarised the effect of COVID-19 in patients with schizophrenia, and the potential mechanisms which may underly poor COVID-19 prognosis in people with schizophrenia. The current evidence suggests that people with schizophrenia may be at increased risk of mortality and morbidity from COVID-19. Although this reduced life expectancy was thought to be due to coexisting comorbidities such as CVD, DM and hypertension, all these studies have adjusted for the aforementioned comorbid conditions, as well as other prognostic factors such as age, sex and race. This suggests that schizophrenia “*per-se*” may have an inherent pathophysiological association with COVID-19. Although the exact mechanisms of the poor prognosis is yet to be fully elucidated, there are tentative clues which suggest that this is likely to be multifactorial due to the syndromic nature of schizophrenia (Figure 1). However, a closer look at the data suggests a more complex picture, as the majority of studies did not factor in various other relevant social determinants such as deprivation, poverty and homelessness, all of which are more common in people with schizophrenia, and are also associated with worse outcomes in COVID-19. Therefore, thoughtful assessment of the various factors that contribute to poor COVID-19 prognosis in people with schizophrenia will be crucial to improved patient care.

These emerging observations may have implications for future research and for the management of people with schizophrenia. First, with respect to research, predictive instruments for those most at risk of severe outcomes should consider schizophrenia. For instance, in UK, the National Health Service uses a risk prediction algorithm based on age, ethnicity, deprivation, body mass index, and a range of comorbidities including severe mental illness not just limited to schizophrenia, known as the QCOVID (118). Second, a clearer understanding of the complex pathophysiological mechanisms linking COVID-19 with schizophrenia may provide insights for: (i) clinicians in patient management; (ii) developing preventive measures against COVID-19; and (iii) developing effective therapeutic strategies to potentially reduce the risk of developing severe COVID-19 illness. Furthermore, to facilitate early intervention for COVID-19, more research is warranted to investigate the DDIs between potential COVID-19 treatments and anti-psychotics. Finally, the present definition of ‘clinically extremely vulnerable’ for vaccine prioritisation, does not include patients with SMD (119). Therefore, taking into account of the higher vulnerability to severe COVID-19 infections and increased mortality in people with schizophrenia, it is paramount that COVID-19 vaccine allocation should also be prioritised for this group of patients.

Our knowledge of the public health consequences of SARS-CoV-2 infection in patients with schizophrenia at this early stage is limited. Clinical management of COVID-19 in this group of patients may pose a clinical challenge to clinicians. Further research is needed from resource-limited settings such as low-and-middle-income countries (LMIC) to fill knowledge gaps about the epidemiology, clinical characteristics, incidence, mortality and morbidity of COVID-19 in people with schizophrenia. There is a paucity of COVID-19 outcome data from LMICs where health inequalities and treatment gaps are much wider than high income countries. The epidemiological studies included in this article were primarily conducted in high income countries, thus limiting the clinical observations generalisable to LMICs. Moreover, the majority of the LMICs have a younger population with a high prevalence of cardiometabolic diseases compared to high income countries, which makes the effects of pandemic even more dire. Finally, the bidirectional relationship between COVID-19 and psychiatric disorder (18) should bolster further research to elucidate the mechanisms by which SARS-CoV-2 (e.g., neurotropism) might contribute to psychiatric sequela. Previous studies from prospective birth cohorts have shown longitudinal associations between inflammation and psychosis (120–122), suggesting that abnormal inflammation may increase the risk of psychosis. Findings from Mendelian randomization also support a potential causal role of inflammation on schizophrenia pathogenesis and comorbid schizophrenia (123).

Of note, we acknowledge the limitations of this hypothetical article. First, given the heterogeneity of the literature and the broad research question we have sought to tackle, a systematic review or meta-analytic approach was not possible. Second, given the large volume and rapid nature of COVID-19 research, we have discussed and cited data from preprint reports on bioRxiv along with peer-reviewed manuscripts. Therefore, these findings should be interpreted with caution. Third, the data on COVID-19 and schizophrenia are derived from non-randomised, observational studies, and we recognise the inherent limitations of such studies. However, the large sample sizes of some studies and robust statistical methods used (case-control studies, propensity score–matched cohort analyses etc), may have helped limit the potential biases and residual cofounding influences. Finally, evidence about SARS-CoV-2 is rapidly evolving, therefore the findings of this article might vary as the understanding of SARS-CoV-2 progresses.

In conclusion, it appears that SARS-CoV-2 infections are not just confined to cardio-respiratory system, but can also be linked to neuropsychiatric diseases. The findings discussed in this article indicate that people with schizophrenia may confer an inherent risk for COVID-19 related morbidity and mortality, irrespective of underlying comorbidities. Therefore, the notion that people with schizophrenia will already be covered by current vaccine rollout strategies due to their coexisting comorbidities is highly debatable. Given the lack of proven treatment for COVID-19 and with efficacious COVID-19 vaccines now available, we recommend that this group of patients should be prioritised to receive COVID-19 vaccines. In this article, we have provided a snapshot of potential pathophysiological links between SARS-CoV-2 pathogenesis and schizophrenia, which may increase the risk of more severe outcomes. We propose that such closely interlinked prognostic components must certainly not be overlooked. To meet the urgent need for effective treatment, an integrated public health approach must be made by researchers globally, to investigate and report, the clinical and pathophysiological underpinnings that drive this clinical deterioration. Finally, emerging evidence suggest an increased incidence of neuropsychiatric complications following COVID-19, including mental disorders such as psychosis. Therefore, special attention should be given to the psychiatric aspects of COVID-19.

## Data Availability Statement

The original contributions presented in the study are included in the article/supplementary material, further inquiries can be directed to the corresponding author/s.

## Author Contributions

MM conceived the idea, designed the figure, table and was responsible for synthesising the evidence, and wrote the first draft of the manuscript. MM and BIP develop the search strategy and conducted the literature search. PS and SPS contributed to literature search and added further references. BIP, PS, and SPS critically reviewed and revised the manuscript. All authors read and approved the final manuscript.

## Conflict of Interest

The authors declare that the research was conducted in the absence of any commercial or financial relationships that could be construed as a potential conflict of interest.

## References

[1] Healthmap. Novel coronavirus (COVID-19). Boston: Computational Epidemiology Group. Available online at: https://www.healthmap.org/covid-19/ (accessed March 30, 2021).

[2] Sakurai A Sasaki T Kato S Hayashi M Tsuzuki SI Ishihara T. Natural history of asymptomatic SARS-CoV-2 infection. N Engl J Med. (2020) 383:885–6. 10.1056/NEJMc2013020 32530584 PMC7304419

[3] Zhang X Tan Y Ling Y Lu G Liu F Yi Z. Viral and host factors related to the clinical outcome of COVID-19. Nature. (2020) 583:437–40. 10.1038/s41586-020-2355-0 32434211

[4] Wu Z McGoogan JM. Characteristics of and important lessons from the coronavirus disease 2019 (COVID-19) outbreak in china: summary of a report of 72 314 cases from the chinese center for disease control and prevention. JAMA. (2020) 323:1239–42. 10.1001/jama.2020.2648 32091533

[5] Correll CU Solmi M Veronese N Bortolato B Rosson S Santonastaso P. Prevalence, incidence and mortality from cardiovascular disease in patients with pooled and specific severe mental illness: a large-scale meta-analysis of 3,211,768 patients and 113,383,368 controls. World Psychiatry. (2017) 16:163–80. 10.1002/wps.20420 28498599 PMC5428179

[6] Vancampfort D Correll CU Galling B Probst M De Hert M Ward PB. Diabetes mellitus in people with schizophrenia, bipolar disorder and major depressive disorder: a systematic review and large scale meta-analysis. World Psychiatry. (2016) 15:166–74. 10.1002/wps.20309 27265707 PMC4911762

[7] Vancampfort D Wampers M Mitchell AJ Correll CU De Herdt A Probst M. A meta-analysis of cardio-metabolic abnormalities in drug naïve, first-episode and multi-episode patients with schizophrenia versus general population controls. World Psychiatry. (2013) 12:240–50. 10.1002/wps.20069 24096790 PMC3799255

[8] Zareifopoulos N Bellou A Spiropoulou A Spiropoulos K. Prevalence of comorbid chronic obstructive pulmonary disease in individuals suffering from schizophrenia and bipolar disorder. A systematic review. COPD. (2018) 15:612–20. 10.1080/15412555.2019.1572730 30714418

[9] Perry BI McIntosh G Weich S Singh S Rees K. The association between first-episode psychosis and abnormal glycaemic control: systematic review and meta-analysis. Lancet Psychiatry. (2016) 3:1049–58. 10.1016/S2215-0366(16)30262-0 27720402

[10] Tay MZ Poh CM Rénia L MacAry PA Ng LFP. The trinity of COVID-19: immunity, inflammation and intervention. Nat Rev Immunol. (2020) 20:363–74. 10.1038/s41577-020-0311-8 32346093 PMC7187672

[11] Apicella M Campopiano MC Mantuano M Mazoni L Coppelli A Del Prato S. COVID-19 in people with diabetes: understanding the reasons for worse outcomes. Lancet Diab. Endocrinol. (2020) 8:782–92. 10.1016/S2213-8587(20)30238-2 32687793 PMC7367664

[12] Kozloff N Mulsant BH Stergiopoulos V Voineskos AN. The COVID-19 global pandemic: implications for people with schizophrenia and related disorders. Schizophr Bull. (2020) 46:752–7. 10.1093/schbul/sbaa051 32343342 PMC7197583

[13] Fond G Pauly V Leone M Llorca PM Orleans V Loundou A. Disparities in intensive care unit admission and mortality among patients with schizophrenia and COVID-19: a national cohort study. Schizophr Bull. (2020) sbaa158. 10.1093/schbul/sbaa158 33089862 PMC7665717

[14] Lee SW Yang JM Moon SY Yoo IK Ha EK Kim SY. Association between mental illness and COVID-19 susceptibility and clinical outcomes in South Korea: a nationwide cohort study. Lancet Psychiatry. (2020) 1025–31. 10.1016/S2215-0366(20)30421-1 32950066 PMC7498216

[15] Wang Q Xu R Volkow ND. Increased risk of COVID-19 infection and mortality in people with mental disorders: analysis from electronic health records in the United States. World Psychiatry. (2020) 20:124–30. 10.1002/wps.20806 33026219 PMC7675495

[16] Ji W Huh K Kang M Hong J Bae GH Lee R. Effect of underlying comorbidities on the infection and severity of COVID-19 in Korea: a nationwide case-control study. J Korean Med Sci. (2020) 35:e237. 10.3346/jkms.2020.35.e237 32597048 PMC7324262

[17] Nemani K Li C Olfson M Blessing EM Razavian N Chen J. Association of psychiatric disorders with mortality among patients with COVID-19. JAMA Psychiatry. (2021) 78:380–6. 10.1001/jamapsychiatry.2020.4442 33502436 PMC7841576

[18] Taquet M Luciano S Geddes JR Harrison PJ. Bidirectional associations between COVID-19 and psychiatric disorder: retrospective cohort studies of 62 354 COVID-19 cases in the USA. Lancet Psychiatry. (2020) 8:30–140. 10.1016/S2215-0366(20)30462-4 33181098 PMC7820108

[19] Yao H Chen JH Xu YF. Patients with mental health disorders in the COVID-19 epidemic. Lancet Psychiatry. (2020) 7:e21. 10.1016/S2215-0366(20)30090-0 32199510 PMC7269717

[20] Galea S Merchant RM Lurie N. The mental health consequences of COVID-19 and physical distancing: the need for prevention and early intervention. JAMA Intern Med. (2020) 10.1001/jamainternmed.2020.1562 32275292

[21] Scott KM Lim C Al-Hamzawi A Alonso J Bruffaerts R Caldas-de-Almeida JM. Florescu, de Girolamo G, C. Hu, de Jonge P, Kawakami N, Medina-Mora ME, Moskalewicz J, Navarro-Mateu F, O'Neill S, Piazza M, Posada-Villa J, Torres Y, Kessler RC. Association of Mental Disorders With Subsequent Chronic Physical Conditions: World Mental Health Surveys From 17 Countries. JAMA Psychiatry. (2016) 73:150–8. 10.1001/jamapsychiatry.2015.2688 26719969 PMC5333921

[22] Chafetz L White MC Collins-Bride G Nickens J. The poor general health of the severely mentally ill: impact of schizophrenic diagnosis. Community Ment Health J. (2005) 41:169–84. 10.1007/s10597-005-2651-z 15974497

[23] Filik R Sipos A Kehoe PG Burns T Cooper SJ Stevens H. The cardiovascular and respiratory health of people with schizophrenia. Acta Psychiatr Scand. (2006) 113:298–305. 10.1111/j.1600-0447.2006.00768.x 16638074

[24] Charlson FJ Ferrari AJ Santomauro DF Diminic S Stockings E Scott JG. Global epidemiology and burden of schizophrenia: findings from the global burden of disease study 2016. Schizophr Bull. (2018) 44:1195–203. 10.1093/schbul/sby058 29762765 PMC6192504

[25] Cho SJ Kim J Kang YJ Lee SY Seo HY Park JE. Annual prevalence and incidence of schizophrenia and similar psychotic disorders in the republic of korea: a national health insurance data-based study. Psychiatry Investig. (2020) 17:61–70. 10.30773/pi.2019.0041 31995973 PMC6992854

[26] Benson NM Öngür D Hsu J. COVID-19 testing and patients in mental health facilities. Lancet Psychiatry. (2020) 7:476–7. 10.1016/S2215-0366(20)30198-X 32407671 PMC7213967

[27] Xiang YT Zhao YJ Liu ZH Li XH Zhao N Cheung T. The COVID-19 outbreak and psychiatric hospitals in China: managing challenges through mental health service reform. Int J Biol Sci. (2020) 16:1741–4. 10.7150/ijbs.45072 32226293 PMC7098035

[28] Luykx JJ Lin BD. Are psychiatric disorders risk factors for COVID-19 susceptibility and severity? a two-sample, bidirectional, univariable, and multivariable Mendelian Randomization study. Transl Psychiatry. (2021) 11:210. 10.1038/s41398-021-01325-7 33833219 PMC8027711

[29] Care Quality Commission. COVID-19 Insight 5: Our Data: Care Quality Commission. (2021). Available online at: https://www.cqc.org.uk/publications/major-reports/covid-19-insight-5-our-data (accessed December 20, 2021).

[30] Nasiri MJ Haddadi S Tahvildari A Farsi Y Arbabi M Hasanzadeh S. COVID-19 clinical characteristics, and sex-specific risk of mortality: systematic review and meta-analysis. Front Med. (2020) 7:459. 10.3389/fmed.2020.00459 32793620 PMC7385184

[31] Yang J Zheng Y Gou X Pu K Chen Z Guo Q. Prevalence of comorbidities and its effects in patients infected with SARS-CoV-2: a systematic review and meta-analysis. Int J Infect Dis. (2020) 94:91–5. 10.1016/j.ijid.2020.03.017 32173574 PMC7194638

[32] Williamson EJ Walker AJ Bhaskaran K Bacon S Bates C Morton CE. Factors associated with COVID-19-related death using OpenSAFELY. Nature. (2020) 584:430–6. 10.1038/s41586-020-2521-4 32640463 PMC7611074

[33] Guan WJ Liang WH Zhao Y Liang HR Chen ZS Li YM. COVID-19. Comorbidity and its impact on 1590 patients with COVID-19 in China: a nationwide analysis. Eur Respir J. (2020) 55:2000547. 10.1183/13993003.00547-2020 32217650 PMC7098485

[34] Mehta P McAuley DF Brown M Sanchez E Tattersall RS Manson JJ. HLH across speciality collaboration. COVID-19: consider cytokine storm syndromes and immunosuppression. Lancet. (2020) 395:1033–4. 10.1016/S0140-6736(20)30628-0 32192578 PMC7270045

[35] Müller N Weidinger E Leitner B Schwarz MJ. The role of inflammation in schizophrenia. Front Neurosci. (2015) 9:372. 10.3389/fnins.2015.00372 26539073 PMC4612505

[36] Zhou Z Kang H Li S Zhao X. Understanding the neurotropic characteristics of SARS-CoV-2: from neurological manifestations of COVID-19 to potential neurotropic mechanisms. J Neurol. (2020) 267:2179–84. 10.1007/s00415-020-09929-7 32458193 PMC7249973

[37] Manson JJ Crooks C Naja M Ledlie A Goulden B Liddle T. COVID-19-associated hyperinflammation and escalation of patient care: a retrospective longitudinal cohort study. Lancet Rheumatol. (2020) 2:e594–602. 10.1016/S2665-9913(20)30275-7 32864628 PMC7442426

[38] Myhre PL Prebensen C Strand H Røysland R Jonassen CM Rangberg A. Growth differentiation factor 15 provides prognostic information superior to established cardiovascular and inflammatory biomarkers in unselected patients hospitalized with COVID-19. Circulation. (2020) 142:2128–37. 10.1161/CIRCULATIONAHA.120.050360 33058695 PMC7688084

[39] Song P Li W Xie J Hou Y You C. Cytokine storm induced by SARS-CoV-2. Clin Chim Acta. (2020) 509:280–7. 10.1016/j.cca.2020.06.017 32531256 PMC7283076

[40] Khandaker GM Cousins L Deakin J Lennox BR Yolken R Jones PB. Inflammation and immunity in schizophrenia: implications for pathophysiology and treatment. Lancet Psychiatry. (2015) 2:258–70. 10.1016/S2215-0366(14)00122-9 26359903 PMC4595998

[41] Pillinger T D'Ambrosio E McCutcheon R O DH. Is psychosis a multisystem disorder? A meta-review of central nervous system, immune, cardiometabolic, and endocrine alterations in first-episode psychosis and perspective on potential models. Mol Psychiatry. (2018) 24:776–94. 10.1038/s41380-018-0058-9 29743584 PMC6124651

[42] Upthegrove R Manzanares-Teson N Barnes NM. Cytokine function in medication-naive first episode psychosis: a systematic review and meta-analysis. Schizophr Res. (2014) 155:101–8. 10.1016/j.schres.2014.03.005 24704219

[43] Pillinger T Osimo EF Brugger S Mondelli V McCutcheon RA Howes OD. A meta-analysis of immune parameters, variability, and assessment of modal distribution in psychosis and test of the immune subgroup hypothesis. Schizophr Bull. (2019) 45:1120–33. 10.1093/schbul/sby160 30407606 PMC6737479

[44] Miller BJ Buckley P Seabolt W Mellor A Kirkpatrick B. Meta-analysis of cytokine alterations in schizophrenia: clinical status and antipsychotic effects. Biol Psychiatry. (2011) 70:663–71. 10.1016/j.biopsych.2011.04.013 21641581 PMC4071300

[45] Kumar P Millischer V Villaescusa JC Nilsson IAK Östenson CG Schalling M Ösby U Lavebratt C. Plasma GDF15 level is elevated in psychosis and inversely correlated with severity. Sci Rep. (2017) 7:7906. 10.1038/s41598-017-07503-2 28801589 PMC5554200

[46] Schwieler L Larsson MK Skogh E Kegel ME Orhan F Abdelmoaty S. Increased levels of IL-6 in the cerebrospinal fluid of patients with chronic schizophrenia–significance for activation of the kynurenine pathway. J Psychiatry Neurosci. (2015) 40:126–33. 10.1503/jpn.140126 25455350 PMC4354818

[47] Asadi-Pooya AA Simani L. Central nervous system manifestations of COVID-19: A systematic review. J Neurol Sci. (2020) 413:116832. 10.1016/j.jns.2020.116832 32299017 PMC7151535

[48] Mao L Jin H Wang M Hu Y Chen S He Q. Neurologic manifestations of hospitalized patients with coronavirus disease 2019 in Wuhan, China. JAMA Neurol. (2020) 77:683–90. 10.1001/jamaneurol.2020.1127 32275288 PMC7149362

[49] Tremblay ME Madore C Bordeleau M Tian L Verkhratsky A. Neuropathobiology of COVID-19. The Role for Glia. Front Cell Neurosci. (2020) 14:592214. 10.3389/fncel.2020.592214 33304243 PMC7693550

[50] Hoertel N Sánchez-Rico M Vernet R Jannot AS Neuraz A Blanco C. Observational study of haloperidol in hospitalized patients with COVID-19. PLoS ONE. (2021) 16:e0247122. 10.1371/journal.pone.0247122 33606790 PMC7895415

[51] Kajani R Apramian A Vega A Ubhayakar N Xu P Liu A. Neuroleptic malignant syndrome in a COVID-19 patient. Brain Behav Immun. (2020) 88:28–9. 10.1016/j.bbi.2020.05.042 32425332 PMC7232071

[52] Wang MT Tsai CL Lin CW Yeh CB Wang YH Lin HL. Association between antipsychotic agents and risk of acute respiratory failure in patients with chronic obstructive pulmonary disease. JAMA Psychiatry. (2017) 74:252–60. 10.1001/jamapsychiatry.2016.3793 28055066

[53] Laskaris LE Di Biase MA Everall I Chana G Christopoulos A Skafidas E. Microglial activation and progressive brain changes in schizophrenia. Br J Pharmacol. (2016) 173:666–80. 10.1111/bph.13364 26455353 PMC4742288

[54] Petrilli CM Jones SA Yang J Rajagopalan H O'Donnell L Chernyak Y. Factors associated with hospital admission and critical illness among 5279 people with coronavirus disease 2019 in New York City: prospective cohort study. BMJ. (2020) 369:m1966. 10.1136/bmj.m1966 32444366 PMC7243801

[55] Jegede O Anand Raman A Tiongson B Garlapati PR Hershberger J Gayam V. Clinical characteristics, hospital course, and outcomes among COVID-19 positive patients with mental illness in a community hospital in New York City. Int J Mental Health. (2020) 50:14–5. 10.1080/00207411.2020.1845567

[56] Karpiński P Samochowiec J Frydecka D Sasiadek MM Misiak B. Further evidence for depletion of peripheral blood natural killer cells in patients with schizophrenia: a computational deconvolution study. Schizophr Res. (2018) 201:243–8. 10.1016/j.schres.2018.04.026 29681501

[57] Varchetta S Mele D Oliviero B Mantovani S Ludovisi S Cerino A. Unique immunological profile in patients with COVID-19. Cell Mol Immunol. (2021) 18:604–12. 10.1038/s41423-020-00557-9 33060840 PMC7557230

[58] Zhang R Dong L Shao F Tan X Ying K. Antipsychotics and venous thromboembolism risk: a meta-analysis. Pharmacopsychiatry. (2011) 44:183–8. 10.1055/s-0031-1280814 21739416

[59] Papola D Ostuzzi G Gastaldon C Morgano GP Dragioti E Carvalho AF. Antipsychotic use and risk of life-threatening medical events: umbrella review of observational studies. Acta Psychiatr Scand. (2019) 140:227–43. 10.1111/acps.13066 31264708

[60] Malas MB Naazie IN Elsayed N Mathlouthi A Marmor R Clary B. Thromboembolism risk of COVID-19 is high and associated with a higher risk of mortality: a systematic review and meta-analysis. EClinicalMedicine. (2020) 29:100639. 10.1016/j.eclinm.2020.100639 33251499 PMC7679115

[61] Polat A Çakir U Gündüz N. Leukocytosis after clozapine treatment in a patient with chronic schizophrenia. Noro Psikiyatr Ars. (2016) 53:87–8. 10.5152/npa.2015.9855 28360774 PMC5353246

[62] Marder SR Cannon TD. Schizophrenia. N Engl J Med. (2019) 381:1753–61. 10.1056/NEJMra1808803 31665579

[63] Govind R Fonseca de Freitas D Pritchard M Hayes RD MacCabe JH. Clozapine treatment and risk of COVID-19 infection: retrospective cohort study. Br J Psychiatry. (2020) 1–7. 10.1192/bjp.2020.151 32713374 PMC7417985

[64] Sabe M Dorsaz O Huguelet P Kaiser S. Toxicity of psychotropic drugs in patients with COVID-19: a systematic review. Gen Hosp Psychiatry. (2021) 70:1–9. 10.1016/j.genhosppsych.2021.02.006 33631694 PMC7890235

[65] May M Beauchemin M Vary C Barlow D Houseknecht KL. The antipsychotic medication, risperidone, causes global immunosuppression in healthy mice. PLoS ONE. (2019) 14:e0218937. 10.1371/journal.pone.0218937 31242264 PMC6594635

[66] May M Slitzky M Rostama B Barlow D Houseknecht KL. Antipsychotic-induced immune dysfunction. A consideration for COVID-19 risk. Brain Behav Immun Health. (2020) 6:100097. 10.1016/j.bbih.2020.100097 32835296 PMC7308744

[67] Barbui C Conti V Cipriani A. Antipsychotic drug exposure and risk of venous thromboembolism: a systematic review and meta-analysis of observational studies. Drug Saf. (2014) 37:79–90. 10.1007/s40264-013-0127-6 24403009

[68] Helms J Tacquard C Severac F Leonard-Lorant I Ohana M Delabranche X. High risk of thrombosis in patients with severe SARS-CoV-2 infection: a multicenter prospective cohort study. Intensive Care Med. (2020) 46:1089–98. 10.1007/s00134-020-06062-x 32367170 PMC7197634

[69] Klok FA Kruip JHAM van der Meer NJM Arbous MS Gommers AMP D Kant KM. Incidence of thrombotic complications in critically ill ICU patients with COVID-19. Thromb Res. (2020) 191:145–7. 10.1016/j.thromres.2020.04.013 32291094 PMC7146714

[70] Llitjos JF Leclerc M Chochois C Monsallier JM Ramakers M Auvray M. High incidence of venous thromboembolic events in anticoagulated severe COVID-19 patients. J Thromb Haemost. (2020) 18:1743–6. 10.1111/jth.14869 32320517 PMC7264774

[71] Levi M Thachil J Iba T Levy JH. Coagulation abnormalities and thrombosis in patients with COVID-19. Lancet Haematol. (2020) 7:e438–40. 10.1016/S2352-3026(20)30145-9 32407672 PMC7213964

[72] Ostuzzi G Papola D Gastaldon C Schoretsanitis G Bertolini F Amaddeo F. Safety of psychotropic medications in people with COVID-19: evidence review and practical recommendations. BMC Med. (2020) 18:215. 10.1186/s12916-020-01685-9 32664944 PMC7360478

[73] Association AD Association AP. Consensus development conference on antipsychotic drugs and obesity and diabetes. J Clin Psychiatry. (2004) 65:267–72. 10.4088/jcp.v65n0219 15003083

[74] Sattar N McInnes IB McMurray JJV. Obesity a risk factor for severe COVID-19 infection: multiple potential mechanisms. Circulation. (2020) 142:4–6. 10.1161/CIRCULATIONAHA.120.047659 32320270

[75] Stefan N Birkenfeld AL Schulze MB. Global pandemics interconnected - obesity, impaired metabolic health and COVID-19. Nat Rev Endocrinol. (2021) 17:135–49. 10.1038/s41574-020-00462-1 33479538

[76] Popkin BM Du S Green WD Beck MA Algaith T Herbst CH. Individuals with obesity and COVID-19. A global perspective on the epidemiology and biological relationships. Obes Rev. (2020) 21:e13128. 10.1111/obr.13128 32845580 PMC7461480

[77] Hoffmann M Kleine-Weber H Schroeder S Krüger N Herrler T Erichsen S. SARS-CoV-2 cell entry depends on ACE2 and TMPRSS2 and is blocked by a clinically proven protease inhibitor. Cell. (2020) 181:271–80.e8. 10.1016/j.cell.2020.02.052 32142651 PMC7102627

[78] Richter FC Alrubayyi A Teijeira Crespo A The Oxford-Cardiff COVID-19 Literature Consortium Hulin-Curtis S. Impact of obesity and SARS-CoV-2 infection: implications for host defence - a living review. Oxf Open Immunol. (2021) 2:iqab001. 10.1093/oxfimm/iqab001 PMC7928648 34192269

[79] Ahn SY Sohn SH Lee SY Park HL Park YW Kim H. The effect of lipopolysaccharide-induced obesity and its chronic inflammation on influenza virus-related pathology. Environ Toxicol Pharmacol. (2015) 40:924–30. 10.1016/j.etap.2015.09.020 26509733

[80] Viani-Walsh D Kennedy-Williams S Taylor D Gaughran F Lally J. Vitamin D deficiency in schizophrenia implications for COVID-19 infection. Ir J Psychol Med. (2020) 1–10. 10.1017/ipm.2020.107 32912355 PMC7545242

[81] Mitchell F. Vitamin-D and COVID-19: do deficient risk a poorer outcome? Lancet Diabetes Endocrinol. (2020) 8:570. 10.1016/S2213-8587(20)30183-2 32445630 PMC7239633

[82] Zhang A Leng Y Zhang Y Wu K Ji Y Lei S. Meta-analysis of coagulation parameters associated with disease severity and poor prognosis of COVID-19. Int J Infect Dis. (2020) 100:441–8. 10.1016/j.ijid.2020.09.021 32947052 PMC7490635

[83] Zuo Y Warnock M Harbaugh A Yalavarthi S Gockman K Zuo M. Plasma tissue plasminogen activator and plasminogen activator inhibitor-1 in hospitalized COVID-19 patients. Sci Rep. (2021) 11:1580. 10.1038/s41598-020-80010-z 33452298 PMC7810990

[84] Berger JS Kunichoff D Adhikari S Ahuja T Amoroso N Aphinyanaphongs Y. Prevalence and outcomes of d-dimer elevation in hospitalized patients with COVID-19. Arterioscler Thromb Vasc Biol. (2020) 40:2539–47. 10.1161/ATVBAHA.120.314872 32840379 PMC7505147

[85] Tang N Bai H Chen X Gong J Li D Sun Z. Anticoagulant treatment is associated with decreased mortality in severe coronavirus disease 2019 patients with coagulopathy. J Thromb Haemost. (2020) 18:1094–9. 10.1111/jth.14817 32220112 PMC9906401

[86] English JA Lopez LM O'Gorman A Föcking M Hryniewiecka M Scaife C. Blood-based protein changes in childhood are associated with increased risk for later psychotic disorder: evidence from a nested case-control study of the alspac longitudinal birth cohort. Schizophr Bull. (2018) 44:297–306. 10.1093/schbul/sbx075 29036721 PMC5814944

[87] Föcking M Sabherwal S Cates HM Scaife C Dicker P Hryniewiecka M. Complement pathway changes at age 12 are associated with psychotic experiences at age 18 in a longitudinal population-based study: evidence for a role of stress. Mol Psychiatry. (2019) 26:524–33. 10.1038/s41380-018-0306-z 30635638 PMC6906256

[88] Hoirisch-Clapauch S Amaral OB Mezzasalma MA Panizzutti R Nardi AE. Dysfunction in the coagulation system and schizophrenia. Transl Psychiatry. (2016) 6:e704. 10.1038/tp.2015.204 26731441 PMC5068878

[89] Jönsson AK Schill J Olsson H Spigset O Hägg S. Venous thromboembolism during treatment with antipsychotics: a review of current evidence. CNS Drugs. (2018) 32:47–64. 10.1007/s40263-018-0495-7 29423659 PMC5843694

[90] MHRA. MHRA Public Assessment Report: The Risk of Venous Thromboembolism with Antipsychotics. (2009). Available online at: https://assets.publishing.service.gov.uk/government/uploads/system/uploads/attachment_data/file/852412/Risk_of_venous_thromboembolism_associated_with_antipsychotics.pdf (accessed January 15, 2021).

[91] Carrizo E Fernández V Quintero J Connell L Rodríguez Z Mosquera M. Coagulation and inflammation markers during atypical or typical antipsychotic treatment in schizophrenia patients and drug-free first-degree relatives. Schizophr Res. (2008) 103:83–93. 10.1016/j.schres.2008.03.004 18436434

[92] Nguyen TT Dev SI Chen G Liou SC Martin AS Irwin MR. Abnormal levels of vascular endothelial biomarkers in schizophrenia. Eur Arch Psychiatry Clin Neurosci. (2018) 268:849–60. 10.1007/s00406-017-0842-6 28942562 PMC8023592

[93] Cai HQ Catts VS Webster MJ Galletly C Liu D O'Donnell M. Increased macrophages and changed brain endothelial cell gene expression in the frontal cortex of people with schizophrenia displaying inflammation. Mol Psychiatry. (2020) 25:761–75. 10.1038/s41380-018-0235-x 30214039 PMC7156343

[94] Perry BI Upthegrove R Thompson A Marwaha S Zammit S Singh SP Khandaker Dysglycaemia G. Inflammation and psychosis: findings from the UK ALSPAC birth cohort. Schizophr Bull. (2019) 45:330–8. 10.1093/schbul/sby040 29635418 PMC6403055

[95] Bikdeli B Madhavan MV Jimenez D Chuich T Dreyfus I Driggin E. COVID-19 and thrombotic or thromboembolic disease. Implications for prevention, antithrombotic therapy, and follow-up: JACC state-of-the-art review. J Am Coll Cardiol. (2020) 75:2950–73. 10.1016/j.jacc.2020.04.031 32311448 PMC7164881

[96] Huang I Lim MA Pranata R. Diabetes mellitus is associated with increased mortality and severity of disease in COVID-19 pneumonia - A systematic review, meta-analysis, and meta-regression. Diabetes Metab Syndr. (2020) 14:395–403. 10.1016/j.dsx.2020.04.018 32334395 PMC7162793

[97] Smith SM Boppana A Traupman JA Unson E Maddock DA Chao K. Impaired glucose metabolism in patients with diabetes, prediabetes, and obesity is associated with severe COVID-19. J Med Virol. (2021) 93:409–15. 10.1002/jmv.26227 32589756 PMC7361926

[98] Wang S Ma P Zhang S Song S Wang Z Ma Y. Fasting blood glucose at admission is an independent predictor for 28-day mortality in patients with COVID-19 without previous diagnosis of diabetes: a multi-centre retrospective study. Diabetologia. (2020) 63:2102–11. 10.1007/s00125-020-05209-1 32647915 PMC7347402

[99] Li H Tian S Chen T Cui Z Shi N Zhong X. Newly diagnosed diabetes is associated with a higher risk of mortality than known diabetes in hospitalized patients with COVID-19. Diabetes Obes Metab. (2020) 22:1897–906. 10.1111/dom.14099 32469464 PMC7283710

[100] Grossmann V Schmitt VH Zeller T Panova-Noeva M Schulz A Laubert-Reh D. Profile of the immune and inflammatory response in individuals with prediabetes and type 2 diabetes. Diabetes Care. (2015) 38:1356–64. 10.2337/dc14-3008 25877811

[101] Mzimela NC Ngubane PS Khathi A. The changes in immune cell concentration during the progression of pre-diabetes to type 2 diabetes in a high-fat high-carbohydrate diet-induced pre-diabetic rat model. Autoimmunity. (2019) 52:27–36. 10.1080/08916934.2019.1575820 30776930

[102] Pillinger T Beck K Gobjila C Donocik JG Jauhar S Howes OD. Impaired glucose homeostasis in first-episode schizophrenia: a systematic review and meta-analysis. JAMA Psychiatry. (2017) 74:261–9. 10.1001/jamapsychiatry.2016.3803 28097367 PMC6352957

[103] Perry BI Burgess S Jones HJ Zammit S Upthegrove R Mason AM. The potential shared role of inflammation in insulin resistance and schizophrenia: a bidirectional two-sample mendelian randomization study. PLoS Med. (2021) 18:e1003455. 10.1371/journal.pmed.1003455 33711016 PMC7954314

[104] Grant MC Geoghegan L Arbyn M Mohammed Z McGuinness L Clarke EL. The prevalence of symptoms in 24,410 adults infected by the novel coronavirus (SARS-CoV-2; COVID-19): a systematic review and meta-analysis of 148 studies from 9 countries. PLoS ONE. (2020) 15:e0234765. 10.1371/journal.pone.0234765 32574165 PMC7310678

[105] Beigel JH Tomashek KM Dodd LE Mehta AK Zingman BS Kalil AC. Remdesivir for the treatment of Covid-19 - final report. N Engl J Med. (2020) 383:1813–26. 10.1056/NEJMoa2007764 32445440 PMC7262788

[106] Horby P Lim WS Emberson JR Mafham M Bell JL Linsell L. Dexamethasone in hospitalized patients with Covid-19. N Engl J Med. (2021) 384:693–704. 10.1056/NEJMoa2021436 32678530 PMC7383595

[107] National Institute for Health and Care Excellence. COVID-19 Rapid Evidence Summary: Remdesivir for Treating Hospitalised Patients With Suspected or Confirmed COVID-19. National Institute for Health and Care Excellence (2020). Available online at: https://www.nice.org.uk/advice/es27 (accessed March 11, 2021).

[108] Perantie DC Brown ES. Corticosteroids, immune suppression, and psychosis. Curr Psychiatry Rep. (2002) 4:171–6. 10.1007/s11920-002-0023-8 12003678

[109] Nelson JC Davis JM. DST studies in psychotic depression: a meta-analysis. Am J Psychiatry. (1997) 154:1497–503. 10.1176/ajp.154.11.1497 9356556

[110] McCullough PA Alexander PE Armstrong R Arvinte C Bain AF Bartlett RP. Multifaceted highly targeted sequential multidrug treatment of early ambulatory high-risk SARS-CoV-2 infection (COVID-19). Rev Cardiovasc Med. (2020) 21:517–30. 10.31083/j.rcm.2020.04.264 33387997

[111] Bramante CT Ingraham NE Murray TA Marmor S Hovertsen S Gronski J. Metformin and risk of mortality in patients hospitalised with COVID-19: a retrospective cohort analysis. Lancet Healthy Longev. (2021) 2:e34–41. 10.1016/S2666-7568(20)30033-7 33521772 PMC7832552

[112] Cameron AR Morrison VL Levin D Mohan M Forteath C Beall C. Anti-Inflammatory effects of metformin irrespective of diabetes status. Circ Res. (2016) 119:652–65. 10.1161/CIRCRESAHA.116.308445 27418629 PMC4990459

[113] Plasencia-García BO Rodríguez-Menéndez G Rico-Rangel MI Rubio-García A Torelló-Iserte J Crespo-Facorro B. Drug-drug interactions between COVID-19 treatments and antipsychotics drugs: integrated evidence from 4 databases and a systematic review. Psychopharmacology (Berl). (2021) 238:329–40. 10.1007/s00213-020-05716-4 33410987 PMC7788177

[114] Ma J Hua T Zeng K Zhong B Wang G Liu X. Influence of social isolation caused by coronavirus disease 2019 (COVID-19) on the psychological characteristics of hospitalized schizophrenia patients: a case-control study. Translational Psychiatry. (2020) 10:411. 10.1038/s41398-020-01098-5 33235185 PMC7685525

[115] Hamada K Fan X. The impact of COVID-19 on individuals living with serious mental illness. Schizophr Res. (2020) 222:3–5. 10.1016/j.schres.2020.05.054 32473931 PMC7250778

[116] Higashi K Medic G Littlewood KJ Diez T Granström O De Hert M. Medication adherence in schizophrenia: factors influencing adherence and consequences of nonadherence, a systematic literature review. Ther Adv Psychopharmacol. (2013) 3:200–18. 10.1177/2045125312474019 24167693 PMC3805432

[117] T. R. C. o. Psychiatrists. COVID-19: Providing medication. London (2021). Available online at: https://www.rcpsych.ac.uk/about-us/responding-to-covid-19/responding-to-covid-19-guidance-for-clinicians/community-and-inpatient-services/providing-medication (accessed April 6, 2021).

[118] Clift AK Coupland CAC Keogh RH Diaz-Ordaz K Williamson E Harrison EM. Living risk prediction algorithm (QCOVID) for risk of hospital admission and mortality from coronavirus 19 in adults: national derivation and validation cohort study. BMJ. (2020) 371:m3731. 10.1136/bmj.m3731 33082154 PMC7574532

[119] Department of Health and Social Care. Guidance on Shielding and Protecting People Who Are Clinically Extremely Vulnerable From COVID-19: Definition of Clinically Extremely Vulnerable Groups. (2021). Available online at: https://www.gov.uk/government/publications/guidance-on-shielding-and-protecting-extremely-vulnerable-persons-from-covid-19/guidance-on-shielding-and-protecting-extremely-vulnerable-persons-from-covid-19 (accessed March 21, 2021).

[120] Khandaker GM Pearson RM Zammit S Lewis G Jones PB. Association of serum interleukin 6 and C-reactive protein in childhood with depression and psychosis in young adult life: a population-based longitudinal study. JAMA Psychiatry. (2014) 71:1121–8. 10.1001/jamapsychiatry.2014.1332 25133871 PMC4561502

[121] Metcalf SA Jones PB Nordstrom T Timonen M Mäki P Miettunen J. Serum C-reactive protein in adolescence and risk of schizophrenia in adulthood: a prospective birth cohort study. Brain Behav Immun. (2017) 59:253–9. 10.1016/j.bbi.2016.09.008 27622678 PMC5176002

[122] Perry BI Zammit S Jones PB Khandaker GM. Childhood inflammatory markers and risks for psychosis and depression at age 24: examination of temporality and specificity of association in a population-based prospective birth cohort. Schizophr Res. (2021) 230:69–76. 10.1016/j.schres.2021.02.008 33684738 PMC8224182

[123] Hartwig FP Borges MC Horta BL Bowden J Davey Smith G. Inflammatory biomarkers and risk of schizophrenia: a 2-sample mendelian randomization study. JAMA Psychiatry. (2017) 74:1226–33. 10.1001/jamapsychiatry.2017.3191 29094161 PMC6583386

